# Emergency rabies control in a community of two high-density hosts

**DOI:** 10.1186/1746-6148-8-79

**Published:** 2012-06-18

**Authors:** Alexander Singer, Graham C Smith

**Affiliations:** 1 Food and Environment Research Agency, Sand Hutton, York, YO41 1LZ, UK; 2 Present address: Department of Ecological Modelling, Helmholtz Centre for Environmental Research – UFZ, Permoserstr. 15, 04318, Leipzig, Germany

**Keywords:** Badger, Cross species infection, Epidemiology, Exotic disease, Fox, Model, Multi-host disease, Population control, Simulation, Vaccination

## Abstract

**Background:**

Rabies is a fatal viral disease that potentially can affect all mammals. Terrestrial rabies is not present in the United Kingdom and has been eliminated from Western Europe. Nevertheless the possibility remains that rabies could be introduced to England, where it would find two potentially suitable hosts, red foxes and badgers. With the aim to analyse the spread and emergency control of rabies in this two species host community, a simulation model was constructed. Different control strategies involving anti-rabies vaccination and population culling were developed, considering control application rates, spatial extent and timing. These strategies were evaluated for efficacy and feasibility to control rabies in hypothetical rural areas in the South of England immediately after a disease outbreak.

**Results:**

The model confirmed that both fox and badger populations, separately, were competent hosts for the spread of rabies. Realistic vaccination levels were not sufficient to control rabies in high-density badger populations. The combined species community was a very strong rabies host. However, disease spread within species appeared to be more important than cross-species infection. Thus, the drivers of epidemiology depend on the potential of separate host species to sustain the disease. To control a rabies outbreak in the two species, both species had to be targeted. Realistic and robust control strategies involved vaccination of foxes and badgers, but also required badger culling. Although fox and badger populations in the UK are exceptionally dense, an outbreak of rabies can be controlled with a higher than 90% chance, if control response is quick and follows a strict regime. This requires surveillance and forceful and repeated control campaigns. In contrast, an uncontrolled rabies outbreak in the South of England would quickly develop into a strong epizootic involving tens of thousands of rabid foxes and badgers.

**Conclusions:**

If populations of both host species are sufficiently large, epizootics are driven by within-species transmission, while cross-species-infection appears to be of minor importance. Thus, the disease control strategy has to target both host populations.

## Background

The management of multi-host infections requires that the true one or two species maintenance host(s) are identified [1], which can be difficult even for well-known diseases such as rabies [2], and will depend on the density of both host species. Rabies is a fatal viral disease that affects mammalian carnivores [3] and is a multi-host disease throughout the world [4]. In Europe, the red fox *Vulpes vulpes* (L.) is considered the major rabies reservoir, but the invasive raccoon dog *Nyctereutes procyonoides* (Gray) becomes an important part of the reservoir as its density increases [5] and is the second most common wildlife rabies host in Europe. There are other wildlife mammals that were reported to play a minor role in rabies epizootics in Europe, such as stone martens *Martes foina* (Erxleben) [6]. However, the comparably low salivary titre of virus may inhibit longer chains [3] and references therein]. Also, small numbers of rabid badgers *Meles meles* (L.) were recorded in areas with epizootic fox rabies [7]. The low number of observed cases of rabid badgers is surprising, given the fact that they are highly susceptible to the fox adapted circulating strain of rabies, produce viral titres similar to foxes and suffer from local eradication suggestive of within-species rabies transmission [3]. However, the low badger density in Eastern Europe would impede larger outbreaks in this species [8].

This situation would be different in Great Britain, where badger densities are much higher than in the rest of Europe [3,9-13]. Due to the higher density, badger control measures would very likely be required to manage a rabies outbreak in wildlife in most parts of Southern Britain [8]. The United Kingdom is free of terrestrial rabies and introduction of the disease into wildlife is highly unlikely [14], particularly following the recent successful rabies eradication in neighbouring Western European countries [15,16]. Nevertheless, the particular conditions of high fox and badger densities would fuel a potential outbreak and cause a major epizootic. The British strategy, therefore, is to combat an outbreak as quickly as possible, trying to eliminate the disease before it spreads and establishes in the high-density host reservoir [17]. In the face of two hosts (foxes and badgers), the control measures need to contain the disease in both species because encounters between species and the potential for cross-species infection are common [3,18,19]. Cross infection produces two problems: (i) the numbers of infected animals and the pool of susceptible animals are increased, which causes more intense outbreaks (essentially increasing R_0_), and (ii) the different species traits and behaviour increase the variability in the epidemiological dynamics (making the prediction of control strategies more uncertain) [20,21].

This simulation study analyzes rabies epidemiology and control in the community of hosts. The analysis is focused on the South of England, because this region has both a high fox density (1–2 foxes km^-2^) [22] and the highest badger densities recorded (up to 25 adults km^-2^) [23]. It is also a likely entry point of the virus into the UK, due to high human population density.

It was shown previously that rabies could establish in a pool of two host species, even if each of the host populations were too small to maintain rabies [24]. In such a case, regular cross-species transmission was essential for rabies spread [21]. However, it is unclear how cross-infection of rabies contributes in a community of host species at higher densities. The British situation provides a good basis for a case study, because of its relevance for disease management, but also for the extensive data on the host species populations.

Our study assesses emergency strategies in response to a recently detected outbreak (< 4 weeks). It is often useful to organize emergency control fundamentally different to management of an endemic disease, because of the spatially localized scenario and because of differing control objectives. In particular, the transient dynamics of an emerging disease that involves heterogeneous distributions of hosts requires that temporal and spatial characteristics of control strategies need careful consideration [25]. For this reason, we applied a spatially-explicit model specifically tailored to the Southern English situation of a rabies outbreak in the fox and badger community. Results of this work should give useful indications for the management of other two-host disease outbreaks, including raccoon dog/fox rabies in Eastern Europe or multi-species canid rabies in Africa.

## Results

### Rabies in single species

**Figure 1:**
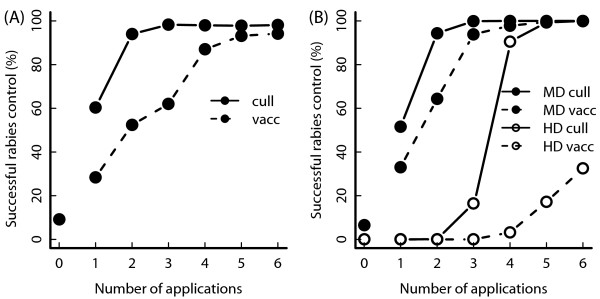
**Success of rabies control in separate hosts.** Success of rabies control in separate hosts: foxes (graph **A**) and badgers (graph **B**, filled circles, medium badger density (MD) as in South East England; open circles: high badger density (HD) as in South West England). In each control application, 40% of animals are treated.

The fox population in the South of England is a competent rabies host. Competent means that the host population can sustain virus transmission such that the disease does not go extinct during the simulated time horizon. In the model, an introduced rabies infection would usually cause an epizootic. Figure 1A shows that rabies can be eliminated from the fox population. Two campaigns of a 40% cull, or five campaigns of a 40% vaccination, are successful in over 90% of simulations.

Badger populations at both medium and high density were also suitable rabies hosts with an epizootic at least as likely in badgers as in foxes (Figure 1B). The success of rabies control in badgers at medium density was similar to that in foxes, although less vaccine applications were required to eliminate rabies. In the high density area, the epizootic was much more intense: disease control required four culling campaigns and six vaccine campaigns were insufficient to eliminate rabies.

### Rabies in host community

**Figure 2:**
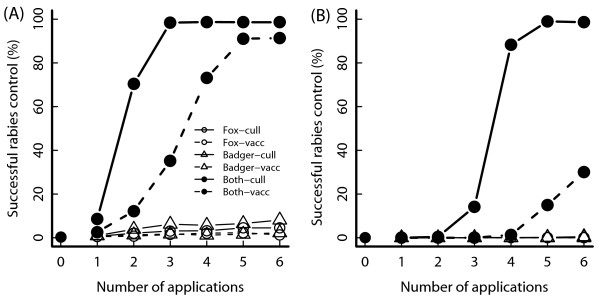
**Success of rabies control in host species community.** Success of rabies control in host community. (Graph **A**: medium badger density in South East England, graph **B**: high badger density in South West England. The fox density is the same in both graphs.) In each control application, 40% of the population of a host species is treated as specified in the legend. Rabies starts in badger population.

As both host populations could sustain rabies, it is not surprising that rabies can spread in the community of species. To control the disease, both species have to be targeted at both medium and high density (Figure 2), as targeting only a single species at best achieved 10% success.

If rabies was controlled in both hosts, control success was comparable to success in that host where rabies was hardest to control. This means that the main effect of cross-transmission was to cause a second self-sustained epizootic, but did not intensify the disease in the other species, nor did it lower control success by re-introducing the virus back into the species in which rabies was eliminated.

The risk of a rabies epizootic was generally slightly lower, if the outbreak occurred initially in foxes. It was found that there was a higher chance (still less than 10%) that a small outbreak in foxes would fade out before badgers were cross-infected.

### Improved control strategy

The previous analysis showed that the risk for development of a wildlife rabies epizootic was high for the South of England. It became clear that at least in high badger density areas, rabies could not be controlled by anti-rabies-vaccination only. Further analysis for the medium density showed that vaccination on its own had difficulty in controlling rabies if realistically achievable vaccination rates were assumed, due to the achievable level in badgers: in the best case scenario (disease outbreak in foxes), the disease was eliminated in no more than 90% of simulations. Therefore, to control rabies in a community of foxes and badgers in the South of England, badgers would have to be culled. We aimed to develop a realistic control strategy under the premise of the lowest possible culling effort.

**Figure 3:**
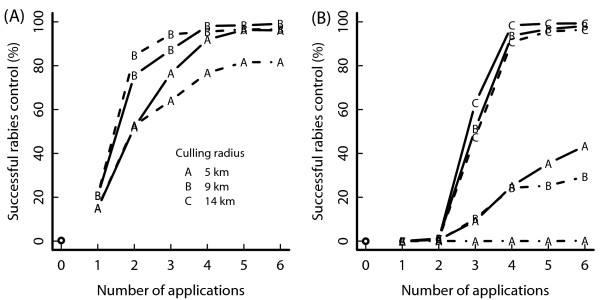
**Success of rabies eradication in host species community.** Success of rabies eradication in species community. The fox density is the same in both graphs. Graph **A**: medium badger density in South East England; graph **B**: high badger density in South West England; control: fox vaccination 70%, badger vaccination 20%, badger culling 40%; varied radius of badger culling area (see legend); dashed line: 6 monthly control application; solid line: 2 monthly control application.

Figure 3 displays a comparison of the success of different control strategies. At medium badger density (Figure 3A), two strategies proved successful: control at a two monthly interval over 4–5 campaigns involving badger culling over an area of 5 km radius. Alternatively, 3–4 campaigns could take place in a 6-monthly interval, but the badger-culling radius would have to be enlarged to 9 km. At high badger density, an even larger badger culling area was required: 9 km for the two monthly intervals and 14 km for the 6 monthly intervals. Both strategies had to be repeated at least 4 times.

### Control effectiveness

**Figure 4:**
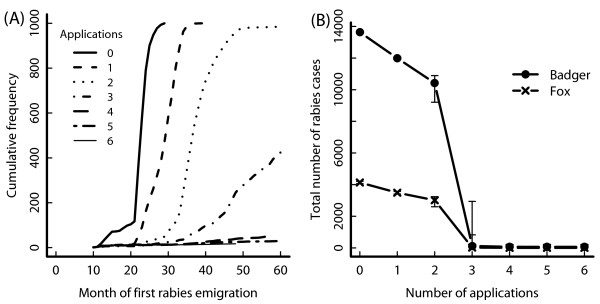
**Disease dynamics of a rabies outbreak with different control applications.** Disease dynamics in high badger density (South West England) in a 38 km x 38 km area over 5 years (rabies starts in month 9); control: 70% fox vaccination, 20% badger vaccination and 40% badger culling within 9 km radius; Results from 1000 simulation repetitions; (**A**) Number of simulation runs in which a rabid animal has already left the simulated area by the month indicated on the x-axis; Different line types indicate the number of repeated applications at two monthly intervals; (**B**) Median and 25% quantiles (error bars) of the accumulated number of rabid animals in the simulated area over the 5 years.

Control affected both the spread and strength of disease (Figure 4). The time until the first rabid animal left the simulated area (i.e. rabies spread beyond the control area) increased with the number of control applications (Figure 4A). Thus, control slowed down the speed of spread of rabies. The fox was primarily responsible for the speed of rabies spread due to its longer dispersal range. However, if the spread was slower, more badgers were involved.

Since rabies first escapes from the simulated area mostly after the last control campaign, it is not surprising that the risk of escape closely matched unsuccessful elimination (Figure 3). Thus, if control ended unsuccessfully, disease recovered in the host and then spread beyond the observed area.

The total number of rabies cases accumulated over the simulated 5-year period was much higher if disease was not eliminated (0–2 applications: Figure 4B). In these cases disease recovered after control finished producing more rabies cases. Nevertheless, the number of cases declined with increasing control effort.

At high badger density, around twice as many rabies cases were predicted in badgers than in foxes, due to differences in population densities. In an outbreak, the higher badger density is a major concern: the model showed a slow spatial spread of rabies through the badger population. As the zone of high infection in badgers moved outwards from the centre, rabies infected almost all animals.

In contrast, if rabies could be eliminated (4–6 applications), in at least 75% of simulation runs, the total number of rabies cases stayed below 100. Hence, the disease was safely controlled.

### Certainty of control strategy success

Sensitivity analysis demonstrated that population density and control measures were important for the success of the control strategies. The model predicted that for badger family sizes with on average up to 10 adults and fox families with an average size of 5, rabies elimination could be achieved with more than 90% certainty. However, control effectiveness must not drop much below that suggested above. For low levels of control per campaign, the model predicted that success rates in high badger density could fall below 70%. Increasing the number of campaigns to six did increase the chance of success but still did not achieve rabies elimination in 90% of the simulations. Further analysis revealed that a low control probability for one species could lead to a problematic drop in the success of control. Control of rabies in badgers was most important, although effective fox vaccination had to be maintained.

Disease transmission rates within or between species were relatively unimportant, due to high host density. In many simulations, if rabies started in foxes, a single cross-transmission event to badgers was sufficient to exacerbate the epizootic. If rabies started in badgers, the problem was severe from the start.

## Discussion

This study looked at emergency disease control in a two-species system, where both species were competent hosts. Previous studies of rabies control in the UK focused on either rabies epizootics in urban fox populations [26] or in rural badger populations, including the effects of landscape heterogeneity [8]. Both took account of the high density host population in England compared to other European countries, concluded that introduction of rabies in the South of England was highly likely to cause an epizootic and that a contingency plan was justified.

### Rabies in single species

This study confirmed previous results on the high risk of rabies spread in the South of England. Both species, foxes and badgers, would be competent rabies hosts, with either species able to be a sufficient reservoir for a rabies epizootic in the short term.

To compare control efficacy, similar control rates were applied to foxes and badgers. In areas of medium badger density, control efficacy was comparable for foxes and badgers, where 60 – 70% of the population had to be culled, or 70 – 80% vaccinated, in order to achieve high disease elimination success rates. Vaccination effort was comparable to previous findings [27], and may be slightly higher than figures used on the continent [28] due to the higher simulated host density. For a given probability of vaccination, disease elimination was more likely in badgers than in foxes, mainly because recruitment of foxes interfered more with control by vaccination. These findings are in line with earlier modelling that suggests that cub productivity (recruitment of susceptibles) is a major reason for the reduced success of vaccination, and that this could in the future be overcome by the use of fertility control [29].

In areas of higher badger density there were much more intense rabies epizootics in badgers, meaning that the susceptible badger population had to be reduced to around 20% to eliminate disease. Vaccination at relatively high rates of 40% per campaign over one year in two monthly intervals was not successful in eliminating the disease, although this effort meant that more than 90% of the existing population in one year was vaccinated.

### Rabies in a community of foxes and badgers

Multi-host pathogens have recently become more prominent for their impact on conservation (cross-infection to endangered species) [30], agricultural livestock (bovine tuberculosis) [31], and their risk to humans (avian influenza) [32]. Rabies is also a multi-host zoonosis, with the red fox seen as the major wildlife host in Europe, but with spill-over to other mammals. The badger is a known spill-over host for rabies in Europe with a small number of cases [5], it is a suspected short-term host [33] and it was argued that badgers could become hosts if their density was higher, such as in the UK [3].

By using computer models we suggest that the badger population in Southern England could sustain a rabies outbreak in the short-term, and thus it was not surprising that the community of foxes and badgers is a strong rabies host. Multi-host reservoirs have been suggested for rabies in Africa [2], northeast Europe [5] and in parts of the United States [34]. This work suggests that at moderate to high density the fox/badger community could also act as a multi-host reservoir.

We found that rabies control is only effective in the multi-host system if both species are targeted, If only one species was targeted, a single cross infection event would trigger uncontrolled spread in the non-targeted population. The finding is similar to suggested rabies control in Finish raccoon dogs and foxes, where both species had to be targeted, even although separately the species could not sustain the disease [24].

In our study, if both species were controlled, rabies elimination success was almost as high as success rates for the single host species that was harder to control. This indicated that no substantial extra effort had to be taken to control rabies in the host community compared to controlling it in each of the two host species separately. Cross-species transmission appeared to be a minor issue. It did not significantly impact on the risk of an epizootic. This phenomenon can be explained by considering the possible contributions of cross infection on rabies epidemiology and how they affected rabies spread in the model:

First, disease establishment could be more likely due to rabies spread in two species. Indeed, it was more likely that the disease died out, when it started in the fox population compared to a start in a high density badger population. This was because the chance of an initial die out of a small infection was higher in the smaller fox families. However, it took only one cross transmission and badgers (with their larger social groups) became the main driver of disease.

Second, the disease could be re-enforced by cross-species spillover. However, the results show that this was unnecessary, as epizootics can occur in each species separately.

Third, there was the chance of re-infection, after control had eliminated the disease from one of the species. This process turned out to be less important, because the virus was re-introduced into a controlled population, which was now too small to sustain an epizootic on its own. Therefore, each re-introduction caused spill-over cases, but did not affect the over-all success of rabies elimination.

Thus, at low rates of cross-species transmission, the impact of cross-infection for the spread of a disease depends on the ability of host species to sustain the disease independently. If populations of both host species are sufficiently large, epizootics are driven by within-species transmission, while cross-species-infection appears to be of minor importance. In contrast, if single host populations are low (less competent hosts), cross-infection is an essential process to sustain the disease [20,21]. This finding is in line with model results for the spread of canine distemper virus in three wildlife species (lions *Panthera leo*, jackals *Canis adustus* and hyenas *Crocuta crocuta*), where with strong interspecific transmission the virus would be driven into the less competent lion population, while at low rates of cross-infection, spill-over from other species caused erratic small scale outbreaks [35]. Notably, due to cross species transmission, the community of two competent host species, jackals and hyenas, acted similar to a single host population doubled in size, although this did depend on transmission rates. Such qualitative differences in disease dynamics, due to cross-infection, should be considered in disease control strategies.

### Feasible rabies control strategy for southern England

Achievable rates of fox vaccination (~70%) are greater than the minimum required for disease control in rural foxes [36]. In contrast, for badgers, the level of vaccination achieved in reality is much lower because anti-rabies vaccine appears to be less effective in immunizing badgers [3]. It was assumed for this study that, when vaccinating foxes, about 20% of the badger population would be vaccinated as well. This level did not control rabies, even in the South East of England (medium badger density). Therefore a successful rabies control strategy in areas of medium to high badger density had to include more pronounced badger population control. In case of a focal rabies outbreak in the UK, the contingency plan permits limited badger culling, potentially using a poison specifically designed for rabies control [17]. Badger control is highly labour intensive, but it is assumed that a campaign could realistically target a minimum of 40% of the badger population. To reduce effort and non-target impact, we reduced the area in which culling had to be performed without compromising the success.

For medium density, assuming that every two months 70% of foxes and 20% of badgers could be vaccinated within a circle of 18 km radius around the centre of the outbreak, and additionally 40% of badgers (independent of vaccination) would be culled in a radius of 5 km around the outbreak, rabies would be eliminated after 1 year with more than 90% probability. A slightly higher success and quicker elimination was achieved if the badger-culling radius was extended to 9 km. It could be argued that the success rate achieved here is higher than would occur in reality, due to the social perturbation of badgers [37], which may be greater than the simulated level of perturbation in the model, but this will be ameliorated to some extent by the assumption of avoiding stochastic fadeout.

To control rabies in at least 90% of simulations for the high badger density, a minimum badger-culling radius of 9 km was needed. Success could be slightly increased by extending the control area, but both strategies would take about 10 months to reach completion.

If control campaign frequency was reduced to twice yearly, the total control effort (number of campaigns) had to be increased substantially, because “temporal refuges” allowed the disease to spread in initially less-intensively targeted host populations, with enhanced numbers of susceptibles between each reproduction period. This suggests that the EU recommended strategy designed to eliminate endemic rabies [38] is likely to be less successful as an emergency control strategy in medium or high density areas.

The model showed that current UK control options tested in this study were suitable to control a rabies outbreak even in high badger and fox density areas, if well-organized and rapid, assuming an early detection of disease. The suggested strategies proved robust: the strategies controlled species under different assumptions on spatial heterogeneity of host density, which indicates that their effectiveness is in general situations insensitive to landscape heterogeneity and specific landscape features. Instead, failure in control at realistic population densities was only found if at least one of the host species was poorly targeted. Thus, proper organization and commitment will likely lead to successful rabies control.

In some local areas with an extremely high badger density, (e.g. Oxfordshire [9] and Woodchester Park [23]), rabies control success may be lower.

In the model, each control campaign contributed to a reduction in the number of rabies cases as well as a reduction in the speed of disease spread. This was important as the model predicted a median of roughly 18,000 rabies cases for an uncontrolled disease within the high density simulated area (South West England) over a 51-month period. This was on average almost 1 rabies case per month per km^2^ with about three out of four cases in badgers. Control reduced this number considerably, in particular during the period when control was applied. That is, most rabies cases in the model occurred after control ended and the disease was not eliminated but could recover. The small number of rabies cases during control also meant that disease spread was slowed down. Therefore the risk that the virus would escape the controlled area was considerably reduced during the period of control.

Prolonging the period of control improved the chance of eradication success and simultaneously reduced the strength of disease. Thus, even if rabies is not eliminated within the predicted time frame, it is likely that continuing control will achieve elimination, as long as the virus does not escape the control area. Also, given the difficulty of accurately monitoring wildlife disease [39], control should continue after the last recorded case, in accordance with international recommendations.

This study suggests that a rabies outbreak in the South of England can be controlled before it causes a major epizootic. But, if immediate control was not successful, then disease control purely based on anti-rabies vaccination of foxes may not be sufficient to contain and eliminate the disease, and large-scale badger culling is logistically difficult. This suggests that new methods for population control, such as immuno-contraceptives, could be investigated to substitute for culling. A combined application of contraception and vaccination to control fox rabies proved to be about as effective as culling in a simulation study [27]. However, even if such methods become available, the best option for rabies control in the UK and other rabies-free areas will be to prevent introduction of the virus and quickly detect and control any potential disease outbreak.

The case of rabies in Southern England is an important example of the general impact of several large and competent host populations on the spread of disease. Our findings on emergency control strategies for the UK can inform the development of other rabies contingency management e.g. in Northern and Eastern Europe, given the increase of the raccoon dog population, and also for Northern America [40,41].

## Conclusion

In a rabies community of multiple host species emergency control subsequent to a recent rabies outbreak has to contain and eliminate the disease in both species. If several species are competent rabies hosts on their own, within species transmission drives disease spread. The risk of cross-species transmission is that it seeds a new, parallel and relatively independent outbreak in the second species.

The rabies control strategy for a community of wildlife hosts must take into account host-specific behaviour (e.g. movement range, group structure and seasonality of reproduction) and species-specific control effectiveness (e.g. bait uptake and effectiveness of vaccines). Control strategies might involve a range of control actions.

Considering these factors feasible strategies were developed to control a rabies outbreak in the combined high density populations of red fox and badger in Southern England.

## Methods

### Model for rabies spread in foxes and badgers

An individual based stochastic spatial simulation model was written in Visual Basic, based on two previous models for rabies spread in urban foxes [26] and for rabies spread in badgers [8]. The specialty of this new model is that disease dynamics in two host species can be simulated. The model description focuses on the newly included mechanisms and changes that are related to the combination of the two single species rabies models. Nevertheless, we briefly describe all processes in the model. For the detailed description we refer to published literature.

To structure the methods section we follow a standard protocol for the description of individual-based models [42].

### Purpose of the model

The model was constructed to assess options for the emergency control of rabies in Southern England that would commence immediately (< 2 weeks) after the detection of an outbreak of rabies in the host community of foxes and badgers. For this purpose, the model spatio-temporally tracks host populations and virus spread.

### State variables and scales

Two host species were modelled. For each species a single population was considered, structured into family groups. Animals within a group were characterized by their age, sex, health-status and recent movements. For foxes, two ages were distinguished: cubs (during their first year) and adults [8,26]. For badgers, an additional juvenile stage was considered, for their second year, since these badgers do not reproduce [8]. The disease status of an animal was healthy, infected or vaccinated. An infected animal that became infectious died within the same time step [8,26]. Thus the infectious stage was not tracked explicitly. Animals were also recorded if they had recently moved, to avoid unrealistic multi-dispersal within-year events.

The model was set up on a spatial grid of 76 x 76 cells, each cell representing 500 m x 500 m, which was sufficient to model a rabies emergency control area. Social groups inhabited one or more adjacent cells, to permit heterogeneity in territory size and avoid anisotropies associated with movement on regular grids [43]. Social groups therefore consisted of one or more grid cells, which created a more realistic landscape with different numbers of neighbouring groups for each social group. The time step of the model was one month, which reflects a realistic time scale for control actions.

### Process overview and scheduling

**Figure 5:**
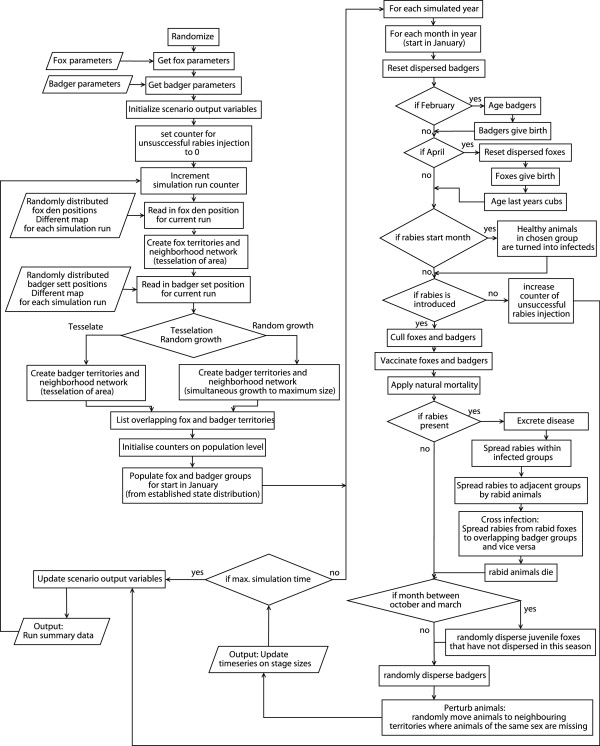
**Flow chart of processes to simulate rabies spread for a single scenario.** The flow chart of the simulation model showing the initial setup on the left, and the annual and monthly loops on the right.

Figure 5 displays the process order in the model. The left part contains initialization and result output, while the process model is depicted on the right. All modelled processes consider seasonality of host populations (reproduction, survival and dispersal) and disease transmission (seasonal infection probabilities). Seasonal processes act only during specified periods of the year (Figure 5) or are parameterized applying seasonally variable parameters (see parameterization, Table 1). Disease management measures follow fixed schedules (Table 2). All actions of one process (represented by a box in Figure 5) were simultaneously updated.

**Table 1:** Population and disease parameters used in the model

**Aggregated parameters**	**Standard value**	**Variation in sensitivity analysis**	**Variation in literature**
Badger mortality (yearly)			
Badger cubs		30 – 53% ^e^	
Male ^a^	47% (seasonal) ^h^	Grouped ^d^	50 – 65% ^g^
Female ^a^	41% (seasonal) ^h^	Grouped ^d^	50 – 65% ^g^
Older than 1year	Cub mortality reduced by 20% to balance badger density		
Male ^a^	16% (seasonal) ^h^	Grouped ^d^	30% ^g^
Female ^a^	12% (seasonal) ^h^	Grouped ^d^	24% ^g^
Fox mortality (yearly)		36 – 53% ^f^	
Cubs male ^a^	53% (seasonal) ^i^	Grouped ^d^	50 – 65% ^g^
Cubs female ^a^	51% (seasonal) ^i^	Grouped ^d^	50 – 65% ^g^
Older than 1year			
Male ^a^	48% (seasonal) ^i^	Grouped ^d^	40 – 60% ^g^
Female ^a^	45% (seasonal) ^i^	Grouped ^d^	40 – 60% ^g^
Fox litter size			
Cubs	4.53 (± 1.54) ^i^	--	
Older than 1 year	4.76 (± 1.53) ^i^	--	
Fox sex ratio (m:f)	0.55 ^i^	--	
Fox: Probability of breeding			
Cubs	0.66 ^i^	--	
Older than 1 year	0.77 ^i^	--	
Badger litter size	2.94 (± 0.94) ^h^	--	
Badger sex ratio (m:f)	0.5 ^h^	--	
Badger: Probability of breeding			
1^st^ female	0.74 ^h^	--	
2^nd^ female	0.37 ^h^	--	
3^rd^ female	0.3 ^h^	--	
Fox dispersal			
Dispersal months	October – March ^i^	--	
Dispersal Distance (range)			
Males	0.6 – 30.8 (density dependent) ^i^	--	
Females	0.6 – 12.6 ^i^	--	
Badger infection probability ^h^			
Within group	0.89 ^h^	0.8 – 1.0 ^b^	20% variation
Between groups		0.8 – 1.2	20% variation
Male - females	0.55 ^h^	Grouped ^c^	
Same sex	0.55 ^h^	Grouped ^c^	
Adult - juveniles	0.19 ^h^	Grouped ^c^	
Fox infection probability			
Within home range	0.999 ^j^	0.8 – 1	20% variation ^b^
Between home ranges		0.8 - 1.1	20% variation ^b^
Juveniles in summer	0.329 ^j^	Grouped ^c^	
Males to females in winter	0.912 ^j^	Grouped ^c^	
All other contacts in winter	0.514 ^j^	Grouped ^c^	
In spring	0.122 ^j^	Grouped ^c^	
In summer	0.499 ^j^	Grouped ^c^	
In autumn	0.255 ^j^	Grouped ^c^	
Fox-Badger cross infection	0.04 ^k^	0.03 –0.05	20% variation
Badger prob. becoming infectious (rabid)	0.42 ^h^	0.3 – 0.5	20% variation
Fox prob. becoming infectious (rabid)	0.42 ^i^	Grouped ^d^	

^a^ Relative seasonality of rates preserved.

^b^ 20% variation intended but limited because parameter would be beyond possible range.

^c^ Varied as group proportional to rate of group parameter.

^d^ Varied as group. Absolute variation range indicated for group parameter.

^e^ Intended 30 – 70% but limitations due to population extinction.

^f^ Intended 30 – 70% but limitations due to minimal seasonal mortality rate and population extinction.

^g^[44].

^h^[8]**(mortality differs between the first two months of life and older).**

^i^[26]**(mortality differs between years one and older, and varies in each month).**

^j^[27].

^k^ see text for derivation of value.

**Table 2:** Rabies control scenarios simulated

**ID**	**Description**	**Control method**	**Controlled Species**	**Efficacy (%)^1^**	**Radius (km) ^2^**	**Timing ^3^ (monthly)**
1	Standardized	Culling or	Single species or	40	18	2
	control	vaccination	both species			
2	Achievable	Vaccination	Both	Fox: 70	18	2 or 6
	vaccination			Badger: 20		
3	Improved	Culling +	Both	Fox	18 (vacc)	2 or 6
		Vaccination		Vacc: 70		
				Badger		
				Vacc: 20		
				Cull: 40	5, 9, 14 (cull)	

^1^ The chance that an animal was treated in a campaign.

^2^ The distance from the initial focal introduction.

^3^ Each campaign was assumed to be instantaneous and occurred at either two-monthly or six-monthly intervals.

Simulation of rabies spread in two host populations was performed by allowing disease to spread between the two species (cross infection), with a local density-dependent symmetrical transmission process. The model ignored competition between host populations (their population dynamics were independent), although we know this is not strictly correct [45]. However, in case of a rabies outbreak with high mortality in both host populations and therefore reduced population densities, it can be assumed that the impact of competition is low.

We explain all model processes briefly in their inner-annual order (right hand side of Figure 5) in the sub-model section. More detailed descriptions of the processes can be found in the cited literature.

### Design concepts

#### Interaction

Within social groups, reproduction was density dependent. Disease transmission was local, through contacts of animals within their social group and with animals of adjacent or overlapping groups.

#### Stochasticity

All processes were stochastic at the individual level.

### Initialization

The flow chart (Figure 5) shows model initialization in the upper left: in each simulation run, spatial fox den and badger main sett locations were randomly generated from predefined densities. A capacity was randomly assigned to each badger sett indicating the maximum number of breeding females. Fox and badger territories were allocated around the fox dens and badger setts by tessellation, assigning each cell to the nearest relevant den/earth. Territories could not overlap that of the same species, but could overlap that of the other species, and remained static during a simulation run. Badger territories could not exceed 9.6 km^2^, so in some low density simulations, badger territories were not completely contiguous. This reduced contiguity was not sufficient to change the disease dynamics [8].

Each group (fox and badger) was then randomly initialized with a stable stage distribution. The stable badger distribution was taken from previous simulations [8]. Initial values for the fox population were calculated from the mean of the last 30 years of 25 repetitions of the model run with standard parameters but without disease. From these simulation results, fox families were initialized in the following way: The family size was calculated from a rounded normal distribution with mean 3.7 and standard deviation 0.075 (truncated at 0). Sexes were randomly attributed with a sex ratio of 0.53 (males). However, if more than one animal was in the group, there was at least one male and one female. Animals were randomly attributed as juveniles with a probability of 0.46 for males and 0.43 for females. This initialization started population dynamics in an established state. Rabies was then injected into one group of one species in the grid centre, during the first simulated September (9^th^ simulation month), which is just prior to the start of juvenile fox dispersal.

The initialization procedure generated random fox and badger populations with spatially heterogeneous densities. Generally underlying to spatially heterogeneous animal distributions are specific landscape characteristics such as elevation or urbanization. Landscape features are therefore considered implicitly in this study. In this sense, the different initial settings cover a broad range of potential landscapes, in which a rabies outbreak could take place. Testing effectiveness of disease control strategies on a set of different landscapes shows their general applicability. In specific situations, particular landscape structures might, however, confound disease control, for example if they support long-distance dispersal of hosts. Such particularities are not explicitly considered in this study.

### Submodels

#### Reset dispersed badgers

Each badger was allowed to move only once a year, but in any time-step (see below), so moved animals were marked. This step removed the mark and enabled badgers to move in the following year.

#### Age badgers

All cubs and juveniles were transferred into age classes juveniles and adults respectively.

#### *Badger birth*

Depending on family hierarchy and group size a variable number of adult females produced litters of up to five offspring [8].

#### Reset dispersed foxes

All foxes that have moved, and were marked to avoid repeated dispersal, were allowed to move again.

#### *Fox birth*

Families with at least one male and one female fox could produce one litter of cubs of random sex. Adult females bred in preference to juveniles and infertility rates were applied [27].

#### Age last year’s cubs

Fox cubs became adults.

#### Introduce rabies

All animals in one group of one host species in the grid centre were moved to the infected stage. This assumption reflects the strong interaction between animals of one group, such that the initial case likely infects all its family members. With this mode of disease introduction we also avoid simulating an epidemic that dies out of its own accord, and to this extent it may be considered a worst case scenario.

#### *Cull foxes and badgers*

Each animal in the designated area was randomly removed from the population with the respective probability [27], as described by the management strategy (species, culling probability, culling period, spatial extent).

#### *Vaccinate foxes and badgers*

Each healthy animal in the designated vaccination area was randomly moved to the vaccinated stage with the respective vaccination probability, as described by the management strategy (species, vaccination probability, vaccination period, spatial extent). Vaccination (and culling) is assumed to involve baiting or trapping, and will thus require the same effort in each campaign to reach a similar proportion of individuals in each campaign [27].

#### *Apply natural mortality*

Each animal was randomly removed from the population at the respective mortality probability. This differed for males, females, juveniles and adults, and also by season of the year [8,26].

#### *Become rabid*

Each infected animal became infectious according to incubation probability [8,26].

#### *Spread rabies within groups*

Each infectious animal within a family group infected all healthy (not vaccinated) family members with the respective within-group infection probability [8,26].

#### *Spread rabies to adjacent groups by rabid animals*

Each infectious animal could randomly infect healthy (not vaccinated) animals of the same species of directly adjacent families, by age class, sex and seasonality dependent infection probabilities [8,26].

#### Cross infection

Each infectious animal could randomly infect healthy (not vaccinated) animals of the other species in family groups of overlapping territories, by a cross-infection probability.

#### Rabid animals die

All infectious animals were removed from the system.

#### *Disperse juvenile foxes*

Dispersal of young foxes occurs in autumn and winter [46]. In the model, juvenile fox dispersal lasted from October to March. Young foxes were randomly chosen to disperse with a sex dependent probability. Each fox could disperse only once during the entire dispersal season with dispersal distance following a truncated negative exponential curve. Minimum and maximum distances depended on fox density (density in their own and adjacent groups) and sex of dispersers [8,26,27].

#### *Disperse badgers*

Badgers rarely disperse further than adjacent territories in high-density areas [47], so each adult or yearling could disperse to adjacent territories with a sex-dependent probability [8].

#### *Perturb animals*

Social perturbation describes movement of animals to neighbouring territories, that is additional to dispersal, and that may enhance their reproduction success [26,48]. Perturbation may enhance rabies transmission in foxes [49] and is known to enhance bovine tuberculosis transmission in badgers [50]. In the model, non-breeding animals of both host species could move to adjacent territories, where animals of the same sex were currently missing. The order of moving animals depended on age within the original family and was implemented as deterministic process.

### Model parameterization

Model parameterization followed from previous models [8,26,27,48,51]. A summary of model parameters is listed in Table 1, with some parameters adjusted for rural populations (see below).

### Fox density

The previous model for fox rabies in the UK concentrated on high-density urban areas [8,26]. Here we adjusted the populations to reflect a rural scenario. In rural Britain, fox densities range from 0.025 pairs/km^2^ in the hills of Scotland up to around 1 group/km^2^ in Wales and Southern England [44]. We used a value of 0.75 fox groups/km^2^ to reflect densities across Southern England (e.g. the New Forest, with a density of 0.76 groups/km^2^[52]). To reflect this density, fox mortality rates in the model were adjusted to give an average disease-free pre-breeding population density of slightly below 3 foxes/group (~ 2.25 foxes/km^2^) (Table 1).

### Badger density and territory distribution

Badger density in Britain is highly variable with adult densities ranging from less than 1–2 km^-2^ to more than 20 km^-2^ and averaging around 10 km^-2^[44]. Here, we looked at two densities: medium density comparable to the English South East (0.25 groups per km^2^ and 6 adults per group) and high density comparable to the South West (0.75 groups per km^2^ with 8 badgers per group). This gives average densities of 1.5 adults per km^2^ in the medium density area and 6 adults per km^2^ in high density.

At high density, badger territories were assumed to be contiguous, while at low densities the assumption on contiguity was relaxed. For population dynamics, non-contiguity means that the number of neighbouring territories was lower, leading to reduced animal movement.

### Cross infection

Based on sensitivity analysis, we used a cross infection probability of 0.04 that a rabid animal infected susceptible animals of the other species, symmetrical between species. The value was chosen pragmatically as the sensitivity analysis showed that the exact value of cross-infection was relatively unimportant. It only had to be ensured that the virus could spread at least once between hosts (see below).

### Model analysis

#### Simulation experiments

To improve disease management, we simulated the different control options, assuming that rabies control started two months after a disease outbreak that occurred in September. We considered both culling and vaccination. To understand the effects of control, we assumed that the control techniques could be applied species-specifically to both populations separately. Both control area and timing could be specified. Every control strategy was applied between one and six times and in each campaign, a percentage of the population was randomly treated (species-specific) in circular areas around the outbreak. The different scenarios are displayed in Table 2. To compare rabies risk and control effectiveness in foxes, badgers and the community of both, a standardized 40% probability of control was applied (see Table 2 - ID1). However, available evidence suggests that badger vaccination is not as effective as fox vaccination [3], so a low value (20%) was used for badgers in other scenarios.

### Output statistics

We analysed disease risk, strength and spread over the course of five simulated years from 1000 repeated simulation runs. Disease risk was calculated as the percentage of simulations in which the disease was present (persisted in the simulation area or had reached the edge) after five years. Disease strength was defined as the accumulated number of rabid animals in the simulated area during five years. The speed of disease spread was assessed from the time it took until the disease first appeared at the edge of the simulated area. These metrics were calculated separately for each species.

### Sensitivity analysis

The effect of cross infection was assessed by varying the cross infection probability over the range from 0 to 0.1. Further, a full sensitivity and uncertainty analysis [53] was performed for the best control strategies. We used the Bayesian emulator method GemSA [54], available from http://www.tonyohagan.co.uk/academic/GEM/index.html, that, in a previous test (unpublished information), performed well for aggregated model output of individual-based simulation models. To improve interpretation, we grouped parameters that logically belonged together (e.g. monthly mortality rates). The sensitivity analysis comprised population, epidemiological parameters and disease control parameters (Table 1).

Mortality rates of both species were used to effectively vary population density in a disease-free population (although, this could not be done without influencing turn-over rate). Disease-free population sizes were calculated for equidistant points over the range of mortality rates (as described above), taking into account badger group size. Population sizes were then interpolated by spline-fits. The disease-free population sizes were also used to adjust initial population sizes. Thus, for all varied mortality rates, the model started with established disease-free populations. An important part of the sensitivity analysis was to assess impact of control effort: The radius of fox and badger culling was varied between 14 and 19 km, the radius of badger culling between 7 and 12 km. The probability of fox vaccination, badger vaccination and badger culling were ±20% of the rates in the default control strategy.

## Competing interests

The authors declare that they have no competing interests.

## Authors’ contributions

GCS made substantial contributions to the concept and design of the study and original programs. AS performed substantial re-programming, ran the simulations and was primarily responsible for the interpretation. Both authors drafted the manuscript and approved the final version.
